# Conceptual framework for emotion control integrating empathy, emotional regulation, and flexibility

**DOI:** 10.3389/fpsyg.2026.1753106

**Published:** 2026-10-27

**Authors:** Le Roy Barbara, Del Cul Antoine, Trousselard Marion, Ramdani Céline

**Affiliations:** 1 Human Adaptation Institute (HAI), Marseille, France; 2 Hôpital La Pitié-Salpêtrière, Paris, France; 3 French Armed Forces Biomedical Research Institute, Brétigny-sur-Orge, France; 4 Ecole de Psychologues Praticiens (EPP), Paris, France; 5 APEMAC/EPSAM, Metz Cedex, France; 6 University of Lorraine, Inserm, INSPIIRE, Nancy, France; 7 French Armed Forces Biomedical Research Institute, Toulon, France

**Keywords:** cognitive empathy, coping flexibility, emotion control, emotion regulation, executive function

## Abstract

Emotions play an active role in the decision-making process and must be controlled to promote “good decisions” and avoid “bad decisions.” Emotions can be controlled using emotional regulation. Emotional regulation needs the perception of our own and other people’s emotions. The perception of other’s emotion relies on empathy. Empathy and emotional regulation are closely related. When the emotional regulation strategy fails, flexibility enables the implementation of another emotion regulation strategy. We propose a framework of emotion control integrating empathy, emotional regulation, and flexibility. On-going emotion is supervised, and online emotion control takes place. Online emotion control is evidenced by empathy and emotion regulation processes. When the emotional regulation strategy needs to be changed because it did not trigger the appropriate behavior, the offline emotion control takes place. Offline emotion control is evidenced by coping flexibility. We assume that emotion control processes are supported by the prefrontal cortex and are part of executive functions. 166 volunteers completed questionnaires testing empathy (Interpersonal Reactivity Index), emotional regulation (Cognitive Emotion regulation Questionnaire), and coping flexibility (Coping Flexibility Scale). Our results showed a close relationship between cognitive empathy, emotional regulation, and coping flexibility, supporting our framework.

## Introduction

According to the neuroscientist Antonio Damasio (2000), emotions emerge through the perception of our body (a bottom-up process), and this process is fundamental to their rise into our consciousness. Emotions emerge from an interaction between bottom-up (ascending sensory/visceral data) and top-down (expectations, goals, and internal models) processes within the amygdala–insula–cingulate–prefrontal circuits (Comte et al., 2016). These changes in the body and brain related to emotional processes guide behavior and decision-making. In fact, according to Bechara and Damasio (2005), “emotions are a major factor in the interaction between environmental conditions and human decision processes”. The decision-making process involves considering different options and selecting the one most likely to achieve the desired outcome (Byrnes, 2002). Emotions play an active role in the decision-making process and can support the reasoning process.

### Emotion felt and emotion control

According to the emotion-imbued choice (EIC) model (Lerner et al., 2015), emotions permeate decision-making at several levels: the emotion felt *at the moment* of the decision and *the expected emotion*. Here, emotions operate as potent, widespread, and predictable influences on decision-making, sometimes detrimental and sometimes advantageous (Lerner et al., 2015; Phelps et al., 2014). In situations where emotions are harmful drivers of decision-making, they can interfere with reasoning and result in inappropriate behavior: emotions are out of synchronization with the situation and “take over” (Yu, 2016). To circumvent such circumstances, it is imperative that these emotions be effectively regulated and controlled, thereby averting the formation of unfavorable decisions. The concept of emotional control can be conceptualized in a manner analogous to that of cognitive control (Figure 1). As cognitive systems are required to perpetually monitor actions and implement adaptive measures with the objective of enhancing task performance and averting future errors (Mattes et al., 2022), emotional control systems are designed to continuously monitor emotional states to prevent erroneous decisions and undesirable behaviors (Koch et al., 2018; Roelofs et al., 2023). Just as motor action and the control of action are two distinct processes, so too are the emotion felt and the control of emotions (Cisler and Olatunji, 2012).

**Figure 1:**
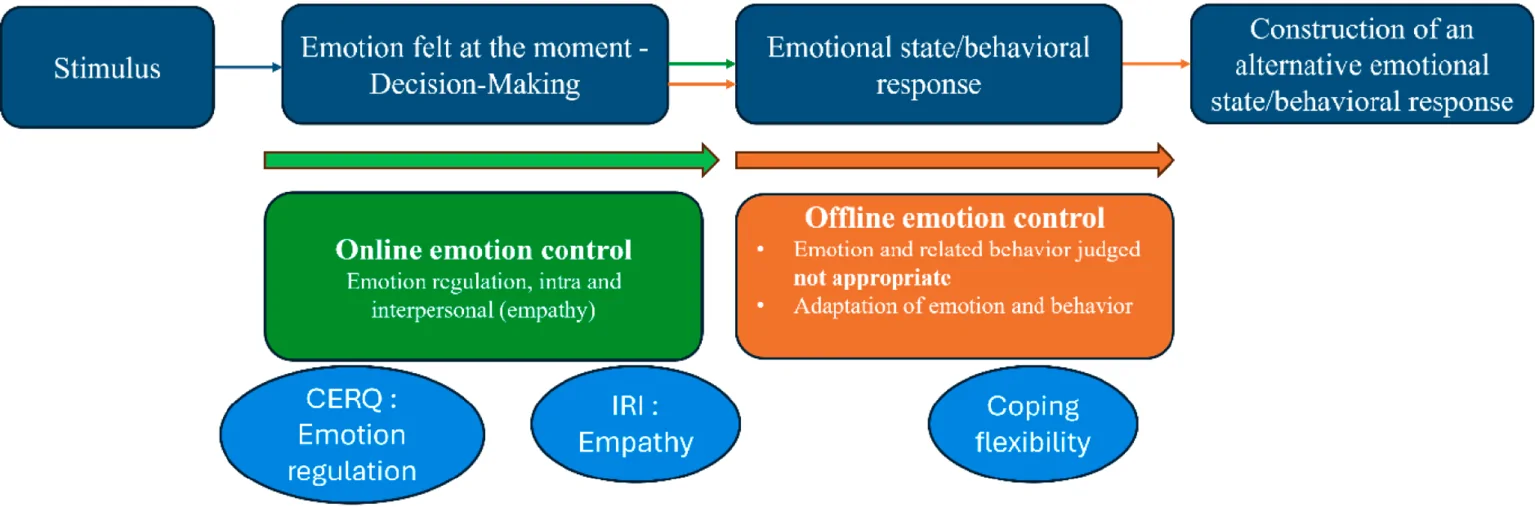
Model of emotion control. When a stimulus appears, an emotion is triggered and influences decision-making. This emotion is controlled by online emotional control (green box). Both intra- and interpersonal (cognitive empathy) emotional regulation processes are activated. If online emotional control fails to promote the appropriate emotional state and behavioral response, offline emotion control takes over after the event. Thanks to coping flexibility, alternative emotional and behavioral responses are developed to fit the situation.

### Online and offline emotion control

Emotion control is implemented whenever an emotion arises, to monitor and correct the emotion and its related behavior when they are inappropriate (Koch et al., 2018; Roelofs et al., 2023). We suggest incorporating emotional control, as outlined by Koch et al. (2018) and described as the Flexible Emotion Control Theory by Roelofs et al. (2023), into a unified dynamic emotion control model.

Emotional control is deployed in parallel of emotion felt. It can regulate the on-going emotion, as it is felt in the moment, it is what we’ll call “online” emotion control. Online control corresponds to emotional control according to Koch, for whom emotion control refers to the regulation of emotions (Koch et al., 2018).

Another aspect of emotional control is proposed by Roelofs et al. (2023), the Flexible Emotion Control Theory. Flexible Emotion Control Theory is a neurocognitive theory that proposes that the ability to regulate emotions effectively depends on the capacity to rapidly switch between alternative strategies, while simultaneously evaluating the current strategy and unchosen counterfactual options. This is particularly important when demands are changing and there is time pressure. The Flexible Emotion Control Theory complements previous aspects of emotion control which considers emotional regulation in terms of predictions, prediction errors, and valuation (“good for me” or “bad for me”) (Etkin et al., 2015) and the ability to flexibly switch between different emotional control strategies (Koch et al., 2018). As this aspect of emotional control takes place after the emotional event, we will refer to it as “offline” emotional control.

We sustain the existence of a set of processes involved in emotional control and part of the executive functions, supported by the prefrontal cortex. Prefrontal cortex regions serve key cognitive functions related to social and emotional aspects of behavior (i.e., switching from one emotion control strategy to another). Furthermore, emotional regulation, cognitive empathy, and coping flexibility share common brain structures (Roelofs et al., 2023; Koch et al., 2018; Etkin et al., 2015).

### Emotional regulation as a marker of online emotion control

We assume that emotional regulation (as measured by self-questionnaires such as the cognitive emotion regulation questionnaire) could be a marker of emotion control which monitors the emotion during the behavior in progress, when the emotion is felt at the moment of the decision (in reference to the EIC model of Lerner et al., 2015).

Emotional regulation (also described as emotional control, according to Koch et al., 2018) can be defined as “the set of automatic and controlled processes involved in the initiation, maintenance, and modification of the occurrence, intensity, and duration of feeling states (Gross and Thompson, 2007)”.

Emotional regulation is a set of processes whose purpose is to increase or decrease the frequency, intensity, or duration of emotional states by acting on their physiological, cognitive, and behavioral components. Through this, individuals shape their emotions and the way they experience and express them (Gross, 2014). Emotional regulation comprises strategies that alter attention, meaning, or responses to shape emotional experience and behavior, consistent with Gross’s process model distinguishing antecedent-focused (before emotion fully unfolds) and response-focused (after) regulation (McRae and Gross, 2020). There are various strategies that can be used to regulate emotions: moving attention away from the stimulus (e.g., through distraction), reassessing how you feel (e.g., through cognitive reappraisal) and changing the way you act in response to what you feel (e.g., through emotional action control). Continuous monitoring and adjustments are required for effective emotional regulation or “online” emotional control, as it is an ongoing process (for a review see: Roelofs et al., 2023).

Emotional regulation is here understood as an intrapersonal emotion regulation, and emotion regulation can also be an interpersonal process (Nozaki and Mikolajczak, 2020): when people make an active effort to change others’ emotions (Trujillo et al., 2022) or when a person applies strategies chosen by another person (Levy-Gigi and Shamay-Tsoory, 2017). Emotional regulation is useful in interpersonal relationships and conflict management. Interpersonal emotional sharing relies on empathy.

### Empathy as a marker of online emotion control

Empathy can be defined as the ability to experience and understand what others feel without confusion between oneself and others (Decety and Lamm, 2006). Empathy is considered an affective function (bottom-up processing) that permits individuals to focus intuitively and automatically on the emotions of others and experience them. Empathy is also a cognitive and conscious function (top-down) that allows individuals to differentiate others’ emotions from their own and to understand someone’s perspective by considering differences with their own perspective (Berthoz and Jorland, 2004; De Vignemont and Singer, 2006). Top-down processing relies on and is affected by executive functions (Cai and Qi, 2023). Many authors therefore propose distinguishing empathy according to an affective component and a cognitive component (Davis, 1980; Preston and De Waal, 2002; Decety and Jackson, 2004; Singer et al., 2006; Birnie et al., 2010; Davis et al., 2018). The ability to identify our emotions and those of others appears to be a key component of adaptive emotional regulation (Gross, 2014).

### Relationship between emotional regulation and empathy

Empathy and emotional regulation seem intrinsically related. Several previous theories have proposed a close relationship between empathy and emotion regulation (e.g., Decety, 2010; Schipper and Peterman, 2013; Thompson et al., 2019; Zaki, 2014). It seems that this close relationship refers more to cognitive empathy than to “global” empathy. In a recent paper, Thompson et al., (2022) showed that high cognitive empathy was broadly associated with better emotion regulation skills, while high affective empathy was typically associated with increased difficulties in emotional regulation. We propose that cognitive empathy, as an interpersonal emotion regulation process, could be another marker of online emotion control. However, affective empathy is not expected to become a third marker of emotional control because it is mostly a bottom-up process. In our proposal, emotional control, as cognitive control, is an executive function. Emotional regulation and empathy are state-like processes. The decisions we make, and our resulting behavior, may be appropriate one day and inappropriate the next. We must adapt our coping strategies of emotional regulation to an ever-changing environment. This ability refers to offline emotional control in our proposal.

### Coping flexibility as marker of offline emotional control

We proposed that coping and flexibility ability, measured by the coping flexibility scale (CFS) (Kato, 2012), could be markers of the implementation of offline emotional control.

Coping is defined as “the set of constantly changing cognitive and behavioral efforts deployed to manage specific internal and/or external requirements that are assessed (by an individual) as consuming or exceeding their resources” (Lazarus and Folkman, 1984). On the one hand, internal demands can be one’s own unpleasant emotions, and coping here would refer to intrapersonal emotional regulation. On the other hand, external demands can be other people’s unpleasant emotions, and coping here would refer to cognitive empathy. The ability to interrupt an ineffective coping strategy and develop and implement an alternative coping strategy is coping flexibility (Kato, 2012). When a person encounters a stressful emotion, they will try to solve the problem. However, the coping strategy (emotional regulation strategy or cognitive empathy skills) used may not always produce favorable outcomes. If the person continues to use an ineffective coping strategy, the situation is unlikely to improve and may worsen. Therefore, evaluation coping occurs when a person begins to abandon the coping strategy that produces undesirable outcomes. However, the person needs to not only abandon the ineffective strategy but also consider using an alternative coping strategy, if needed. This process is termed adaptive coping, and it involves strategies such as creating available alternatives, as well as implementing them.

In summary, we integrate models of Koch et al. (2018) and the FECT (Roelofs et al., 2023) into a dynamic emotion control model. We assume that emotional control is initially deployed when an emotion arises. This is what we call “online” emotional control. We suggest that the markers of its implementation should be emotional regulation (as measured by the CERQ, i.e., intrapersonal emotion regulation) and cognitive empathy ability (interpersonal emotion regulation). If online emotional control has failed to properly regulate the emotion at hand, a shift in emotional regulation or cognitive empathy strategies is needed. Emotion control still monitors the situation and allows the switch of strategy to cope with the discomfort of the situation. This is what we call “offline” emotional control, as it occurs after the fact or event. We proposed that coping flexibility could be a marker of the implementation of offline emotion control.

The aim of this study was to initially highlight individual differences in these markers and concepts and how they covaried within this model. We asked healthy neurotypical volunteers (neurotypical is a non-medical term that describes people whose brains develop and work like most people’s brains do; https://my.clevelandclinic.org/health/articles/neurotypical) to complete self-report questionnaires to evaluate the relationship between these different components of this dynamic emotional control.

*Emotional regulation* was evaluated by the cognitive emotion regulation questionnaire (CERQ) (Garnefski et al., 2001). Both *cognitive and affective empathy* were evaluated by the Interpersonal Reactivity Index (IRI) (Davis, 1983). The IRI evaluates four dimensions of empathy in assessing affective and cognitive components: perspective taking (PT) assesses the spontaneous tendency to adopt the point of view of others; empathic concern (EC) assesses the tendency to experience someone else’s feelings and to feel sympathy or compassion for an unhappy person; fantasy (FS) assesses the tendency to imagine oneself in a fictitious situation; and personal distress (PD) assesses the tendency to feel the distress of others. PD seems to align more closely with anxiety rather than empathy (Murphy et al., 2020), and a researcher who developed the IRI suggested that FS does not theoretically measure empathy (Davis, 1994). It measures the tendency to imaginatively transpose oneself into fictional situations. A previous study showed that a two-factor model with EC and PT exhibited superior model fit parameters compared to other factor models (Wang et al., 2020). Furthermore, many researchers believe that EC is a core factor of emotional empathy, while PT is a core factor of cognitive empathy (e.g., Davis et al., 1987) Therefore, we focused on EC and PT as markers of affective empathy and cognitive empathy, respectively. *Coping flexibility* was evaluated by the coping flexibility scale (CFS) (Kato, 2012). We expected a strong relationship between emotional regulation, cognitive empathy, and coping flexibility processes.

## Materials and methods

### Design

The study was mainly conducted at Institute of Biomedical Research of Army (IRBA) in France over a nine-month period. This is an exploratory study that focuses on collecting psychometric data from a cohort of 166 voluntary. This study has been approved by the Ethics Committee of Paris-Descartes University and all participants provided written informed consent.

We applied Fisher’s transformation of the expected correlation coefficient ρE (with a two-tailed test at *α* = 0.05 and a power of 80%; i.e. *β* = 0.20). In psychology, published correlations show percentiles ~0.17 (median) and 0.33 (75th), suggesting testing scenarios 0.20–0.30 (Weinerová et al., 2022). Testing was conducted for two values of ρE: 0.20 and 0.30. This resulted in a sample size of between 85 and 194 subjects.

### Participants

166 voluntary neurotypical participants (85 men and 81 women) between the ages of 18 and 60 years (*M =* 32.05; *SD =* 11.98) and whose mother tongue was French were recruited via posters placed within the School of Practitioner Psychologists (EPP), IRBA, and the Higher School of Industrial Physics and Chemistry (ESPCI). Ultimately, 65 students at the EPP, 90 workers at the IRBA, and 11 executives and students at the ESPCI responded. The participants were neurotypical subjects affiliated to a university without any neurological, psychiatric, or endocrine pathology. Their medical history was free of any stroke or transient ischemia, as well as head trauma with loss of consciousness or concussion. The use of B-Blocker and other drugs influencing the functioning of the autonomic nervous system was checked to avoid any interference in the study.

### Procedure

Each volunteer completed a series of questionnaires for characterizing a socio-demographic profile, an empathy profile, and an ability to regulate emotions, on a Windows laptop computer, model Acer PREDATOR Helios 300 PH315-51-58KC.

### Socio-demographic questionnaire

A questionnaire developed by the start-up Open Mind Innovation, composed of fifteen items, collects standard socio-demographic data such as age, sex, origin, marital status, level of education, height, weight, laterality of hand and eye as well as characteristics of the participant’s environmental context (professional and personal environment).

### Interpersonal reactivity index (IRI) to assess empathy profile

The IRI questionnaire is composed of twenty-eight items, nine of with are reversed in the French version (Davis, 1983. French tradition: Gilet et al., 2013) and rated on a Likert scale of 1 to 7, ranging from « does not describe me well » to « describes me very well ». The French version of the IRI has shown evidence of good reliability and convergent validity with related constructs. It was designed to evaluate empathy tendencies in adults across four dimensions, assessing both affective and cognitive components. The first, perspective taking (PT), assesses the spontaneous tendency to adopt the point of view of others. Empathic concern (EC) assesses the tendency to experience someone else’s feelings and to feel sympathy or compassion for an unhappy person. Fantasy (FS) assesses the tendency to imagine oneself in a fictitious situation and personal distress (PD) assesses the tendency to feel the distress of others. We focused on EC (marker of affective empathy) and PT (marker of cognitive empathy) and investigated their relationship with emotion regulation.

The Cronbach alpha coefficient was calculated to judge the internal consistency qualities. An acceptable level of internal consistency is indicated when it is greater than 0.70 (Cronbach, 1951; Bland and Altman, 1997). This coefficient indicates an acceptable level for the IRI questionnaire with 0.74.

### Cognitive emotional regulation questionnaire (CERQ) to assess emotional regulation abilities

Garnefski and his collaborators (2001) developed a questionnaire, the Cognitive Emotional Regulation Questionnaire (CERQ), which measures cognitive emotional regulation strategies. This questionnaire was translated into French and validated in 2006 (Jermann et al., 2006). The CERQ is a multidimensional questionnaire composed of 36 items and rated on a Likert scale of 1 to 5, ranging from « almost never » to « almost always ». It was designed to bring out cognitive processes and strategies for regulating cognitive emotions (or cognitive coping strategies) used by an individual after experiencing negative life events. Unlike other adaptation questionnaires that do not make an explicit distinction between an individual’s thoughts and actual actions, this questionnaire refers exclusively to an individual’s thoughts after experiencing a negative event. The CERQ assesses what participants tend to think after experiencing threatening or stressful life events across nine dimensions: « Acceptance», «Positive refocusing», «Refocus on planning», «Positive reappraisal», «Putting into perspective», «Self-blame », «Rumination», «Catastrophizing», and «Blaming others». “Acceptance” refers to thoughts of accepting what you have experienced and resigning yourself to what has happened. “Positive refocusing” refers to thinking about joyful and pleasant issues instead of thinking about the actual event. “Refocus on planning” refers to thinking about what steps to take and how to handle the negative event. “Positive reappraisal” refers to thoughts of creating a positive meaning to the event in terms of personal growth. “Putting into perspective” refers to thoughts of brushing aside the seriousness of the event/ emphasizing the relativity when comparing it to other events. “Self-blame” refers to thoughts of putting the blame for what you have experienced on yourself. “Rumination” refers to thinking about the feelings and thoughts associated with the negative event. “Catastrophizing” refers to thoughts of explicitly emphasizing the terror of what you have experienced. “Blaming others” refers to thoughts of putting the blame for what you have experienced on the environment or another person. The Cronbach alpha coefficient was calculated to judge the internal consistency qualities. This coefficient indicates a satisfactory level with 0.80. For the French validated version, Cronbach’s alpha is from 0.68 to 0.87 (Jermann et al., 2006).

Although cognitive emotional regulation strategies are related, they are independent of each other. Consequently, classifying them as more or less adaptive can be problematic in global statistical analysis (Carvajal et al., 2022). Therefore, we elected to study these factors independently but to utilize these groupings in our reasoning.

### Coping flexibility scale

The CFS is a questionnaire composed of 10 items, two of which are reversed and rated on a Likert scale of 0 to 3, ranging from “not applicable” to “very applicable.” A French version of the CFS was used (Traber et al., 2022). This version consists of seven items that describe stress-coping situations and are rated on a Likert scale of 1 to 4, ranging from « not applicable » to « very applicable». It was designed to assess coping flexibility defined as the ability to discontinue an ineffective coping strategy (i.e., evaluation coping) and produce and implement an alternative coping strategy (i.e., adaptive coping). This definition encompasses two reciprocal processes, considered as dimensions in this questionnaire: evaluation coping (three items) and adaptive coping (four items).

The Cronbach alpha coefficient was calculated to judge the internal consistency. This coefficient indicates a satisfactory level with 0.86.

### Statistical analysis

Statistical analyses were performed using STATISTICA 13.2. Since the responses to the questionnaires did not respect the normality hypothesis (based on the Kolmogorov–Smirnov test and Lilliefors correction), non-parametric tests were carried out to explore potential differences between the dimensional scores: the Kruskal-Wallis test (for age group as a between-subject factor) and the Mann–Whitney test (for gender group as a between-subject factor). The correlations were analyzed. Correlations refers to Spearman’s ρ, which is a type of correlation that does not require the data to be normally distributed. To account for the increased risk of Type I error associated with multiple comparisons, the false discovery rate was controlled using the Benjamini–Hochberg procedure. Adjusted p -values were reported, and statistical significance was determined using a threshold of *α* = 0.05 following correction.

## Results

The level of empathy was higher in women than in men (Table 1). Perspective taking (PT), empathic concern (EC), personal distress (PD), and fantasy were all higher in women than in men (women: PT = 38.37 ± 1.36, EC = 41.05 ± 1.02; men: PT = 36.02 ± 1.45, EC = [value] ± 1.25; PT: *Z* = 2.37, *p* < 0.05; EC: *Z* = 4.41, *p* < 0.001; PD: *Z* = 4.10, *p* < 0.001; Fantasy: *Z* = 3.95, *p* < 0.001). We had no missing data, and the differences observed can be explained solely by gender in these analyses.

**Table 1:** IRI scores by subcategories by gender, Mann–Whitney *U* test.

Gender	Personal distress factor	Empathic concern factor	Perspective taking factor	Fantasy factor
Female	26.65 ± 1.80	41.05 ± 1.02	38.37 ± 1.36	36.54 ± 1.71
Male	21.26 ± 1.73	37.07 ± 1.25	36.02 ± 1.45	30.99 ± 2.05
*p* value	<0.001	<0.001	<0.05	<0.001

Each scale has been tested individually with gender as between-subject difference.

Fantasy factor score (Table 2) was highest in the youngest group (36.24 ± 1.66) compared to the middle (30.88 ± 3.34) and oldest (32.02 ± 2.57) groups (*H* = 9.58, *p* < 0.01). However, this difference was observed only between the youngest group and the other two groups, namely the middle-aged group and the older group (*H* = 5.67, *p* < 0.05 and *H* = 7.42, *p* < 0.01, respectively). No statistical difference was found between the middle-aged group and the oldest group (*H* = 0.28, *p* = 0.866). No statistical difference was found for the three other factors (PT, EC, or PD) between the three groups of age (PT: *H* = 3.01, *p* = 0.221; EC: *H* = 2.78, *p* = 0.249; PD: *H* = 2.48, *p* = 0.288). No statistical differences were reported between the two age groups for these three factors (18–25/26–40: PT: *H* = 1.14, *p* = 0.285; EC: *H* = 2.88, *p* = 0.09; PD: *H* = 0.004, *p* = 0.948; 18–25/41–60: PT: *H* = 2.75, *p* = 0.10; EC: *H* = 0.61, *p* = 0.435; PD: *H* = 2.58, *p* = 0.11; 26–40/41–60: PT: *H* = 0.27, *p* = 0.601; EC: *H* = 0.46, *p* = 0.494; PD: *H* = 1.10, *p* = 0.295).

**Table 2:** IRI scores by subcategories by age, Kruskal–Wallis ANOVA by ranks each scale has been tested individually with age as between-subject differences.

Age (years-old)	Personal distress factor	Empathic concern factor	Perspective taking factor	Fantasy factor
18–25	24.55 ± 2.02	39.57 ± 1.28	37.87 ± 1.59	36.27 ± 1.66
26–40	24.56 ± 2.29	38.09 ± 1.56	36.81 ± 1.96	30.88 ± 3.34
41–60	22.17 ± 2.10	38.93 ± 1.74	36.33 ± 1.66	32.02 ± 2.58
*p* value	0.288	0.249	0.221	<0.01

In our study, Cronbach’s alpha was 0.79 for PT and 0.69 for EC.

### Correlations IRI and CERQ

We focused our attention on the PT factor (i.e., cognitive empathy) and the EC factor (i.e., affective empathy) of the IRI questionnaire. All factors of the CERQ were tested. Results of correlations are presented as a matrix (see Table 3) and details are reported (Table 4). PD factors are also reported as a marker of anxiety. It appears that the level of cognitive empathy positively correlates with the five CERQ adaptive strategy items (“Acceptance”: Spearman’s Rho = 0.26, *p* < 0.01, “positive refocusing”: Spearman’s Rho = 0.18, *p* < 0.05, “refocusing on planning”: Spearman’s Rho = 0.28, *p* < 0.001, “positive reappraisal”: Spearman’s Rho = 0.43, *p* < 0.001, and “putting in perspective”: Spearman’s Rho = 0.18, *p* < 0.05) and negatively correlates with “blaming others” (Spearman’s Rho = −0.19, *p* < 0.05; one of the four maladaptive strategy items). However, it also positively correlates with “rumination” (Spearman’s Rho = 0.29, *p* < 0.001; one of the four maladaptive strategy items). Affective empathy level also positively correlates with the “rumination” (Spearman’s Rho = 0.28, *p* < 0.001) item only. It is worth noting that the PD factor of IRI negatively correlates with all CERQ adaptive strategy items and positively correlates with three of the four maladaptive items.

**Table 3:** Correlations matrix IRI and CERQ.

CERQ/IRI	Perspective taking	Empathic concern factor	Personal distress factor	Fantasy factor
Self-blame	0.074	0.127	0.267	0.152
Acceptance	0.263	0.087	−0.180	0.069
Rumination	0.293	0.281	0.227	0.154
Positive refocusing	0.183	0.026	−0.241	−0.043
Refocus on planning	0.282	0.176	−0.314	0.151
Positive reappraisal	0.435	0.162	−0.360	0.208
Putting into perspective	0.183	0.032	−0.347	0.029
Catastrophizing	0.013	0.068	0.304	−0.002
Blaming others	−0.193	−0.131	0.066	−0.063

In red, correlations with *p* < 0.05 for two-tailed. In violet, maladaptive strategies and in green: adaptive strategies of CERQ.

In sum, the level of cognitive empathy is mainly related to adaptive emotion regulation strategies, and it correlates with more aspects of emotion regulation than the level of affective empathy (see Tables 3, 4; perspective taking is the marker of cognitive empathy, and empathic concern is the marker of affective empathy). Moreover, as expected (Kamel, 2018) anxiety (represented by the personal distress factor of the IRI) seems negatively related to emotional regulation adaptive strategies and positively related to maladaptive emotional regulation strategies (Table 4).

### Correlation IRI and CFS

Cognitive empathy, represented by the PT factor of IRI, positively correlates with both evaluation coping (Spearman’s Rho = 0.31, *p* < 0.0001) and adaptive coping (Spearman’s Rho = 0.33, *p* < 0.0001; see Tables 5, 6 for details). Affective empathy, represented by the EC factor of IRI, positively correlates with adaptive coping only (Spearman’s Rho = 18, *p* < 0.0; with evaluation coping: Spearman’s Rho = 0.06, *p* = 0.41). On the contrary, anxiety symptoms, represented by the PD factor of the IRI, negatively correlates with both evaluation coping (Spearman’s Rho = −0.24, *p* < 0.001) and adaptive coping (Spearman’s Rho = −0.30, *p* < 0.0001). Anxiety was negatively correlated with flexibility. Fantasy was not correlated with either evaluation coping or adaptative coping.

**Table 4:** Correlations matrix IRI and CFS.

CFS/IRI	Perspective taking	Empathic concern	Personal distress	Fantasy factor
Evaluation coping	0.306	0.065	−0.248	0.082
Adaptive coping	0.330	0.180	−0.301	0.086

In red, correlations with *p* < 0.05 for a two-tailed.

### Correlation CERQ and CFS

Evaluation coping and adapting coping positively correlate with all of the five adaptive factors (Acceptance (Spearman’s Rho = 0.18, *p* = 0.05; Spearman’s Rho = 0.29, *p* < 0.0001), positive refocusing (Spearman’s Rho = 0.30, *p* < 0.001; Spearman’s Rho = 0.36, *p* < 0.0001), refocus on planning (Spearman’s Rho = 0.42, *p* < 0.0001; Spearman’s Rho = 0.57, *p* < 0.0001), positive reappraisal (Spearman’s Rho = 0.41, *p* < 0.0001; Spearman’s Rho = 0.52, *p* < 0.0001) and putting in perspective [Spearman’s Rho = 0.23, *p* < 0.05; Spearman’s Rho = 0.33, *p* < 0.001)] and negatively correlates with one maladaptative factor [catastrophizing (Spearman’s Rho = −0.19, *p* < 0.05; Spearman’s Rho = −0.22, *p* < 0.01); see Tables 7, 8 for details].

**Table 5:** Correlations matrix CERQ and CFS.

CERQ/CFS	Evaluation coping	Adaptative coping
Self-blame	−0.146	−0.171
Acceptance	0.176	0.289
Rumination	−0.005	−0.011
Positive refocusing	0.305	0.365
Refocus on planning	0.419	0.572
Positive reappraisal	0.410	0.517
Putting into perspective	0.230	0.334
Catastrophizing	−0.192	−0.220
Blaming others	−0.141	−0.060

In red, correlations with *p* < 0.05.

**Table 6:** Details of all correlations between IRI and CERQ: Sample size N = 166, the term t(N-2) refers to the *t*-test statistic used to test the null hypothesis “correlation = 0” and to the fact that it follows a Student’s *t*-distribution with N-2 degrees of freedom.

	Perspective taking				Fantasy				Personal distress				Empathic concern			
	Spearman	t(N-2)	*p*-level	*p*-value ajusted BH	Spearman	t(N-2)	*p*-level	*p*-value ajustée BH	Spearman	t(N-2)	*p*-level	*p*-value ajustée BH	Spearman	t(N-2)	*p*-level	*p*-value ajustée BH
Self-blame	0.074	0.952	0.343	0.384	0.152	1.966	0.051	0.108	0.267	3.545	0.001	0.002	0.127	1.642	0.102	0.207
Acceptance	0.263	3.489	0.001	0.002	0.069	0.887	0.377	0.606	−0.180	−2.338	0.021	0.024	0.087	1.114	0.267	0.401
Rumination	0.293	3.919	0.000	0.000	0.154	1.995	0.048	0.108	0.227	2.985	0.003	0.004	0.281	3.748	0.000	0.000
Positive refocusing	0.183	2.378	0.019	0.025	−0.043	−0.554	0.580	0.725	−0.241	−3.181	0.002	0.003	0.026	0.334	0.739	0.739
Refocus on planning	0.282	3.767	0.000	0.000	0.151	1.951	0.053	0.108	−0.314	−4.231	0.000	0.000	0.176	2.293	0.023	0.104
Positive reappraisal	0.435	6.183	0.000	0.000	0.208	2.718	0.007	0.063	−0.360	−4.943	0.000	0.000	0.162	2.109	0.036	0.108
Putting into perspective	0.183	2.388	0.018	0.025	0.029	0.372	0.710	0.797	−0.347	−4.732	0.000	0.000	0.032	0.413	0.680	0.739
Catastrophizing	0.013	0.161	0.872	0.872	−0.002	−0.025	0.980	0.980	0.304	4.093	0.000	0.000	0.068	0.875	0.383	0.489
Blaming others	−0.193	−2.514	0.013	0.023	−0.063	−0.813	0.417	0.606	0.066	0.848	0.398	0.398	−0.131	−1.693	0.092	0.207

With the Benjamini–Hochberg (BH) correction *α* = 0.05.

**Table 7:** Details of correlations between IRI and CFS: Sample size N = 166, the term t(N-2) refers to the *t*-test statistic used to test the null hypothesis “correlation = 0” and to the fact that it follows a Student’s *t*-distribution with N-2 degrees of freedom.

CFS/IRI	Perspective taking			Personal distress			Empathic concern		
	Spearman	t(N-2)	*p*-level	Spearman	t(N-2)	*p*-level	Spearman	t(N-2)	*p*-level
Evaluation coping	0.306	4.119	0.000	−0.248	−3.284	0.001	0.065	0.828	0.409
Adaptive coping	0.330	4.478	0.000	−0.301	−4.036	0.000	0.180	2.345	0.020

**Table 8:** Details of correlations between CERQ and CFS: Sample size N = 166, the term t(N-2) refers to the *t*-test statistic used to test the null hypothesis “correlation = 0” and to the fact that it follows a Student’s *t*-distribution with N-2 degrees of freedom.

CERQ/CFS	Evaluation coping				Adaptative coping			
	Spearman	t(N-2)	*p*-level	*p*-value ajusted BH	Spearman	t(N-2)	*p*-level	*p*-value ajusted BH
Self-blame	−0.146	−1.894	0.060	0.101	−0.171	−2.228	0.027	0.061
Acceptance	0.176	2.283	0.024	0.050	0.289	3.868	0.000	0.000
Rumination	−0.005	−0.069	0.945	0.945	−0.011	−0.143	0.886	0.886
Positive refocusing	0.305	4.098	0.000	0.000	0.365	5.022	0.000	0.000
Refocus on planning	0.419	5.914	0.000	0.000	0.572	8.929	0.000	0.000
Positive reappraisal	0.410	5.764	0.000	0.000	0.517	7.740	0.000	0.000
Putting into perspective	0.230	3.025	0.003	0.011	0.334	4.545	0.000	0.000
Catastrophizing	−0.192	−2.511	0.013	0.039	−0.220	−2.882	0.004	0.018
Blaming others	−0.141	−1.818	0.071	0.101	−0.060	−0.767	0.444	0.555

It appears that flexibility coping skills are positively correlated to adaptive emotional regulation skills.

## Discussion

### Theoretical framework of emotional control

In accordance with Koch’s emotion control model (2018), we argue for the existence of a set of processes involved in emotional control that should be part of the executive functions, supported by the prefrontal cortex. This study did not aim to directly test the assumption itself but instead, initially, sought to evaluate the relationship between cognitive empathy, affective empathy, cognitive emotional regulation, and coping flexibility as markers of emotional control.

As a reminder, executive functions rely on three components: shifting, updating, and inhibition (Miyake et al., 2000). Online emotional control would supervise emotion when it occurs and allow emotion regulation and cognitive empathy implementation when the emotion takes place. Offline emotional control would refer to the ability to adapt an emotion and its related behavior and modify them when they do not seem appropriate, building up an alternative emotional state and a more appropriate behavior. These skills should evidence emotional control processes. The existence of emotional control is postulated in reference to cognitive control or motor control. Emotional regulation and cognitive empathy allow us to deal with emotions occurring at any given moment and to inhibit the automatic decision-making triggered by the ongoing emotion. A controlled adapted behavior is then implemented. If the decision-making is not appropriate to the situation or the context, it must be adjusted through coping flexibility, the second line of emotional control. Empathy and emotional regulation are related (Thompson et al., 2019) and recent evidence revealed that cognitive empathy was associated with improved emotional regulation whereas affective empathy was associated with increased difficulties in specific aspects of self-reported emotional regulation (Thompson et al., 2022).

### Measurement of empathy and limits of IRI factors

Our results support these findings. In our study, empathic skills were measured by the IRI questionnaire. The scoring method where IRI is aggregated into two factors [cognitive empathy: fantasy factor (FS) + perspective taking factor (PT) and affective empathy: personal distress (PD) + empathic concern (EC)] has been shown to be inadequate in previous studies (e.g., Chrysikou and Thompson, 2016; Wang et al., 2020). Notably, the two factors ascribed to affective empathy often display no correlation (e.g., Chrysikou and Thompson, 2016; Wang et al., 2020). Some researchers suggested that the PD factor is more indicative of anxiety traits than of empathy (Murphy et al., 2020). FS does not theoretically measure empathy (Davis, 1994) but reflects the tendency to imaginatively transpose oneself into fictional situations. Given this evidence, we focused on EC and PT, since many researchers believe that EC is the core factor of emotional empathy, while PT is the core factor of cognitive empathy (e.g., Davis et al., 1987).

Across two Spanish samples, including 2,499 college students and 1,438 adults, the IRI showed acceptable reliability, with Cronbach coefficients ranging from 0.72 for EC to 0.74 for PT (Lucas-Molina et al., 2017). Cronbach’s alpha varies across study populations due to factors related to cultural adaptations and specific clinical contexts (Lucas-Molina et al., 2017; Rajput et al., 2020; Huang et al., 2025). Furthermore, adapted or abbreviated versions of an instrument may have alpha coefficients that differ from those observed in the original version (Diotaiuti et al., 2021). In our study, the Cronbach’s alpha coefficient for PT was 0.79, indicating acceptable internal consistency (Campo-Arias and Oviedo, 2008). The alpha value of 0.69 observed for EC in our study was slightly lower than the values generally reported in the literature; however, it remained broadly consistent with previous findings. This discrepancy may reflect the variability reported across studies, as well as the small sample size of our study.

As expected, women in our population showed higher levels of empathy than men, both for affective and cognitive empathy (Elkin et al., 2021). However, these results may be indicative of a heightened propensity among women to report empathic experiences (Michalska et al., 2013). Furthermore, social expectations and gender-role stereotypes may influence perceptions of empathy. On a large scale, self-report questionnaires demonstrate greater disparities among women than men. Conversely, experimental and physiological measures exhibit minimal or no differences, thereby suggesting a social desirability bias in self-reports (Baez et al., 2017; Pang et al., 2023).

### Cognitive and affective empathy in emotional regulation (CERQ)

Emotional regulation, or online emotion control, was measured by the CERQ. In the CERQ questionnaire, two factors (adaptive control and maladaptive control) were extracted by principal component analysis (Garnefski et al., 2001). However, another previous study (Garnefski and Kraaij, 2007) showed that a model encompassing nine correlated factors was a good-fit model parameter. In our study, we found a positive correlation between PT or cognitive empathy and all five adaptive strategies measured by the CERQ (acceptance, positive refocusing, refocus on planning, positive reappraisal, and putting in perspective). These results are consistent with those of a previous study, which revealed that cognitive empathy was negatively related to overall levels of emotional dysregulation, whereas affective empathy was not (Thompson et al., 2022). However, although the positive correlation between acceptance items (which refer to thoughts of accepting what you have experienced and what has happened, implying awareness of thoughts and emotions) and cognitive empathy aligns with previous studies’ findings, we did not find a correlation between acceptance and affective empathy. According to Thompson et al. (2022), emotional awareness difficulties were lower the higher the individual’s cognitive and affective empathy skills. These findings are consistent with previous studies indicating that individuals with higher levels of empathy (particularly cognitive empathy) tend to be more attuned to their own emotions (Rieffe and Camodeca, 2016).

#### Personal distress, anxiety traits, and maladaptive regulation

Our analysis highlights that the PD factor (subscale measuring the tendency to feel anxious and distressed when witnessing others’ suffering [Vilella et al., 2023; Kang et al., 2025)] was mirrored to PT in term of correlation to emotion regulation strategies. Indeed, PD was negatively correlated to all five adaptive factors (acceptance, positive refocusing, refocus on planning, positive reappraisal, and putting in perspective) and positively correlated to three maladaptive factors (self-blame). If PT, in our model, could be used as a marker of cognitive empathy and PD could be more associated to anxiety symptoms, one could postulate that these two traits could be antagonistic in terms of adaptive emotion regulation. These findings seem to be in line with a study conducted on Chinese nurses during the COVID-19 outbreak (Wang et al., 2021), revealing that more frequently engaging in self-blame, rumination, and catastrophizing, as well as less engagement of acceptance and positive refocusing, were associated with more anxiety or depressive symptoms. Our results also seem in line with the hypothesis proposed by Cisler and Olatunji (2012) that emotion regulation strategies could confer vulnerability to anxiety disorders by potentiating the effect of negative affect. We assume that anxiety symptoms could be associated with emotional dysregulation, although we cannot prejudge the influence of one on the other. It should be noted that the relationship between empathy and emotional regulation has been examined in a number of previous studies using trait questionnaires (e.g., Himichi et al., 2021; Henschel et al., 2020; Lockwood et al., 2014). Efficient emotional regulation is associated with a state of general well-being and better psychological health, while 40–75% of psychopathologies are associated with deficits in emotional regulation.

#### Rumination and its links with empathy

Rumination [“I often think about how I feel about what I have experienced” (McKinnon et al., 2020)] was the only factor that showed a positive correlation with EC, PT, and PD. Rumination is often described as preoccupation, or perseveration, which is a possible indicator of the person’s inability to limit mental processing of stressful experiences or events (Smith and Alloy, 2009). People with more sophisticated self-regulation processes can often reframe the experience and “let it go”. In practical terms, an individual who ruminates remains preoccupied with the problem or feelings they are experiencing, without necessarily taking action to alleviate the consequences (Nolen-Hoeksema et al., 2008). The interdependence between cognitions, emotions, and behaviors is a fundamental aspect of the definition of mental rumination. In the CERQ, rumination refers to the tendency to focus repeatedly and passively on one’s negative emotions, failures, and their consequences following a difficult event; it is classified as one of the so-called maladaptive strategies, along with catastrophizing, self-blame, and blaming others (Garnefski et al., 2009). Nonetheless, a study of 476 adults in remission from recurrent depression showed that the CERQ’s nine-factor structure did not fit the clinical data and the nature of rumination did not seem universally consistent across clinical contexts (McKinnon et al., 2020). This means that the adaptive/maladaptive distinction could not be consistent across populations, which directly undermines the justification for classifying rumination as universally maladaptive. Research on the Ruminative Responses Scale (RRS) has shown that brooding and reflection have opposite effects on depression—with brooding alone capturing the maladaptive aspects of rumination (Hasegawa, 2013). Furthermore, according to, four types of rumination (*brooding*: passive, comparative, and negatively self-focused rumination, being the clearly maladaptive component; *reflection:* purposeful inward thinking used to understand emotions or solve problems, being relatively adaptive; *Intrusive rumination*: involuntary, repetitive thoughts that arise after a stressful event; and *deliberate rumination*: intentional reflection on a stressful event to make sense of it) can be described, making rumination both adaptive and non-adaptive. By combining these four components, the CERQ could over-emphasize the maladaptive nature of the construct as a whole.

It should be noted that rumination is a solitary, self-centered cognitive process. Cognitive empathy and empathy in general are interpersonal processes. When discussing rumination and interpersonal processes, we can refer to the concept of “co-rumination.” Co-rumination can be interpreted as a form of cognitive empathy enacted within an interpersonal relationship—but when taken to extremes, it transforms understanding the other person into a cognitive loop that is costly for both members of the dyad (Tudder et al., 2024; DiGiovanni et al., 2025). In a representative sample of 495 British adults, perspective-taking was a direct and significant predictor of co-rumination (Denson et al., 2026), which makes co-rumination an interpersonal expression of cognitive empathy. The correlation observed between empathy and rumination in our study may, in fact, mirror the broader relationship between empathy and co-rumination.

### Coping flexibility and cognitive empathy

#### Cognitive empathy

In the context of emotional control, we expected a positive relationship between coping flexibility as measured by CFS and cognitive empathy as measured by IRI. Coping can be seen as the process underlying how individuals interact with the ever-changing environment. This notion is defined as «“the set of constantly changing cognitive and behavioral efforts deployed to manage specific internal and/or external requirements that are assessed (by an individual) as consuming or exceeding their resources” (Lazarus and Folkman, 1984). According to Kato (2012), evaluation and adaptation are two meta-coping skills that enable coping flexibility. Coping flexibility is the ability to actively try various coping strategies to deal with stressful life changes (Cheng et al., 2014). When a coping strategy seems ineffective, people with good flexibility in coping skills can change their strategy and try something else. Emotional well-being seems to rely on adaptively coping (Zimmer-Gembeck, 2021). For Kato, coping flexibility requires the ability to evaluate and adapt. We actually found a positive correlation between cognitive empathy and both aspects of coping flexibility (evaluation coping and adaptive coping) measured by CFS (Kato, 2012). In line with our results on PD and emotional regulation, we found an opposite type of relationship between PD and coping flexibility: PD was negatively correlated to both aspects of coping flexibility. As for emotion regulation and affective empathy, EC did not show the same relationship with coping flexibility than PT or cognitive empathy. EC was only correlated to adaptive coping, not to evaluation coping.

#### Affective empathy

Since EC reflects affective empathy, a bottom-up emotional perception, it is not expected to be related to evaluation coping. In fact, in the context of the emotional reactivity Perception-Valuation-Action sequence (Gross, 2014), EC was not expected to allow a valuation of action (“good for me, bad for me”) since valuation implies a top-down evaluation of the emotion. The valuation of emotion is probably needed for the evaluation coping process. Nonetheless, EC is related to adapting flexibility because evaluation coping is not required for adapting coping. Adapting coping can be implemented after an updating of the action resulting from the decision-making. The valuation of the emotion is “shunted.” In this case, EC can be likened to the model-free regulation of Etkin et al. (2015); that is to say, a direct perception-action pathway, shunting valuation stage. On the contrary, PT or cognitive empathy could be likened to the model-based regulation of Etkin et al. (2015), implying all the three stages of the Perception-Valuation-Action sequence. The valuation stage could then be evaluated thanks to the evaluation coping of the coping flexibility scale.

#### Fantasy factor

FS was not related to coping flexibility, but the creativity ability measured by IRI was also not expected to be related to coping flexibility. It seems that creative thought involves dynamic interactions of large-scale brain systems (Beaty et al., 2016). The PFC (*via* the executive network and the default mode network) is involved but on a smaller scale than expected for emotion control. The relationship between FS and emotional control is then less obvious since they only partially share common brain structures.

### Prefrontal cortex and emotional control

The findings of this study lend support to the hypothesis that there exists a close relationship between the scale empathy of the IRI questionnaire, the scale for emotion regulation of the CERQ questionnaire, and coping flexibility skills measured by CFS.

Although this study does not provide evidence, these skills are hypothesized to reflect the development of emotional control within our framework and are expected to be supported by the PFC, as previously suggested by Koch et al. (2018). Indeed, the dorsolateral prefrontal cortex (dlPFC) strongly supports a cognitive empathic response, represented by perspective-taking processes, and emotion regulation (Domínguez-Arriola et al., 2022) and lesions of the dlPFC imply perseveration behaviors (Jones and Graff-Radford, 2021), The dlPFC can mediate emotional responses and regulate emotion *via* projections through the ventro-medial PFC to regulate the output of the subgenual cingulate, which has extensive connections to limbic areas and the brainstem to initiate emotional/visceral responses (Joyce et al., 2024).

The potential existence of emotional control could be evidenced in the brain using error negativity (a response-locked event-related potential generated by the anterior cingulate cortex and the pre-SMA, within the medial prefrontal cortex) (Falkenstein et al., 2000; Bonini et al., 2014). The Ne should be larger in situations where the expected emotion or the emotion regulation implemented is not the required one. These hypotheses remain speculative and should be tested in future research.

### Emotional awareness, metacognition, and executive functions

Cognitive empathy and emotion regulation, as evaluated by the CERQ, refer to the tendency to reflect on one’s own emotions and those of others. These skills refer to the concept of emotional awareness (Smith et al., 2022) and more broadly to metacognition, which is the ability to reflect on our own cognition and mental states (Seow et al., 2021).

The relationship between anxiety traits (PD factor of IRI) and emotional control is coherent with the fact that alterations of neuron activity, synaptic plasticity, and connectivity within the PFC are correlated with high risk for anxiety disorders (Xu et al., 2019). Furthermore, patients with anxiety disorders showed dysfunctional metacognition (Vishwanathan et al., 2022) and reduced emotional awareness (Sendzik et al., 2017). This is consistent with our hypothesis that integrates at different magnifications, the metacognition (the bigger magnification), the emotional awareness (the middle magnification) and the emotion control (the smaller magnification). These propositions should be interpreted cautiously until confirmed by further empirical evidence.

## Conclusion

The proposed framework is dynamic, sequential, and grounded in neurocognitive processes. It suggests that emotional regulation and cognitive empathy function as indicators of an online control system, while coping flexibility represents a secondary, offline process activated when initial regulation fails. The framework is also explicitly tied to executive functions and neural substrates, particularly prefrontal regions and performance-monitoring systems. However, the empirical study relies solely on cross-sectional trait questionnaires analyzed using pairwise Spearman correlations. Although this approach can reveal covariation between individual differences in these constructs, it does not allow for testing temporal dynamics, switching processes, executive monitoring, or underlying neural mechanisms. Consequently, the findings support associations between self-reported traits rather than providing direct evidence for the proposed dynamic model.

In our view, emotional control mechanisms allow for the updating of emotional contextual information and adaptation accordingly. These understandings should be extended to executive functions and to emotional awareness and more broadly to metacognition. Cognitive empathy presents a positive relationship with emotional control, both online emotional control and offline emotion regulation. Anxiety-related tendencies (measured by CERQ) were negatively related to emotional control, both online (emotion regulation) and offline (coping flexibility). Although anxiety scores were not measured directly, we can reasonably assume that developing cognitive empathy skills could help reduce anxiety symptoms and promote emotional control. These preliminary hypotheses merit additional investigation in future work. Perhaps in a reciprocal way, developing emotional control could reduce anxiety symptoms. Further studies are required to test these hypotheses.

## Data Availability

The raw data supporting the conclusions of this article will be made available by the authors without undue reservation.
